# Stimulation of metacyclogenesis in *Leishmania* (*Mundinia*) *orientalis* for mass production of metacyclic promastigotes

**DOI:** 10.3389/fcimb.2022.992741

**Published:** 2022-09-05

**Authors:** Wetpisit Chanmol, Narissara Jariyapan, Kanok Preativatanyou, Chonlada Mano, Pongsri Tippawangkosol, Pradya Somboon, Paul A. Bates

**Affiliations:** ^1^ School of Allied Health Sciences, Walailak University, Nakhonsithammarat, Thailand; ^2^ Center of Excellence in Vector Biology and Vector-Borne Disease, Department of Parasitology, Faculty of Medicine, Chulalongkorn University, Bangkok, Thailand; ^3^ Faculty of Medicine, Chiang Mai University, Chiang Mai, Thailand; ^4^ Department of Parasitology, Faculty of Medicine, Chiang Mai University, Chiang Mai, Thailand; ^5^ Division of Biomedical and Life Sciences, Faculty of Health and Medicine, Lancaster University, Lancaster, United Kingdom

**Keywords:** *Leishmania*, *Leishmania orientalis*, leishmaniasis, metacyclogenesis, metacyclic promastigote, stimulation, cultivation

## Abstract

*Leishmania* (*Mundinia*) *orientalis* is a human pathogen causing leishmaniasis and studies on the properties of metacyclic promastigotes, the parasite’s infective stage, are required for a better understanding of its transmission and infection. However, information on cultivation for mass production of *L. orientalis* metacyclic promastigotes and factors that stimulate their metacyclogenesis is limited. Therefore, the objective of this study was to develop a suitable methodology for generating promastigote cultures containing a high proportion and number of *L. orientalis* metacyclic promastigotes. Various media, i.e., Schneider’s insect medium, Medium 199 and Grace’s insect medium, supplemented with various quantities of dithiothreitol, Basal Medium Eagle vitamins, pooled human urine, and fetal bovine serum, were optimized for metacyclogenesis. The results revealed that the optimum culture medium and conditions of those tested were Schneider’s insect medium supplemented with 100 μM dithiothreitol, 1% (v/v) Basal Medium Eagle vitamins, 2% (v/v) pooled human urine, and 10% (v/v) fetal bovine serum, pH 5.0 at 26°C. We also demonstrated that *L. orientalis* metacyclic promastigotes could be purified and enriched by negative selection using peanut lectin. Under these culture conditions, the highest yield of metacyclic promastigotes was obtained with a significantly higher percentage of parasite survival, resistance to complement-mediated lysis, and infection index in THP-1 macrophage cells compared to parasites cultured without media supplements at neutral pH. This is the first report providing a reliable method for mass production of *L. orientalis* metacyclic promastigotes for *in vivo* infections and other experimental studies of this emerging parasite in the future.

## Introduction

*Leishmania* parasites cause a group of anthropozoonoses called leishmaniases, which are important tropical neglected diseases. The diseases are transmitted to mammalian hosts *via* the inoculation of metacyclic promastigotes by insect vectors (Bates, 2007). The three main forms of leishmaniases are cutaneous (the most common), mucocutaneous and visceral disease. Their clinical spectrum ranges from asymptomatic infection or self-limiting cutaneous lesions to lethal disseminated infections depending on the species of *Leishmania* parasite and the host immune response (WHO, 2021).

In the *Leishmania* life cycle the parasites alternate between amastigotes that reside in mammalian macrophages and promastigotes that develop in the alimentary tracts of their vectors, which are mainly phlebotomine sand flies, but possibly also midges (Becvar et al., 2021). In infected sand flies, several different forms of promastigote have been described including procyclic promastigotes, nectomonad promastigotes, leptomonad promastigotes, metacyclic promastigotes, and haptomonad promastigotes (Rogers et al., 2002; Gossage et al., 2003). Recently, a new form called retroleptomonad promastigotes has been described, a leptomonad-like form originating from retained metacyclic promastigotes after a subsequent blood meal(s) of the infected sand fly (Serafim et al., 2018). However, the differences between leptomonad promastigotes of the first infected blood meal and the retroleptomonad promastigotes in terms of biochemical and physiological properties remains unknown (Bates, 2018).

Metacyclic promastigotes are the mammal-infective stages naturally found in the anterior midgut and stomodeal valve of the sand fly vectors (Rogers et al., 2002). Therefore, understanding the properties of metacyclic promastigotes is important to understanding disease transmission. Although promastigotes have been cultured axenically in a variety of culture media, typically only a small proportion of metacyclic promastigotes can be generated, which accumulate at the stationary phase of growth *in vitro* (Zakai et al., 1998). However, because of their importance as the parasite’s infective stage, usable, and ideally purified, amounts of metacyclic promastigotes are required for the study of their biological, physiological and biochemical properties and for infection studies *in vitro* and *in vivo*. Consequently, several methods have been developed to enrich or purify metacyclic promastigotes from cultures, for example, lectin-based enrichment techniques that exploit differences in surface membrane glycosylation between metacyclic promastigotes and non-metacyclic promastigotes (Saraiva et al., 1986; Pinto-da-Silva et al., 2002) and density gradient centrifugation (Spath and Beverley, 2001; Yao and Asayama, 2017). These methods work to varying degrees of efficiency depending on the *Leishmania* species being used. However, in all cases, enrichment procedures might not be very effective if the initial parasite cultures contain only a low proportion of metacyclic promastigotes, for example, for use in experiments requiring high concentrations such as *in vivo* infection assays where only small injectable volumes can be used, 5-200 μl/mouse depending on the injection route. In that regard, Bates and Tetley (1993) developed a culture technique to induce metacyclogenesis in *Leishmania mexicana* isolated from mice by using acidified culture medium. Relatively homogeneous populations of promastigotes obtained using this method exhibited functional characteristics consistent with metacyclic promastigotes, such as resistance to complement-mediated lysis, possession of a thickened surface membrane coat and a high infectivity index in peritoneal macrophages (Bates and Tetley, 1993).

*Leishmania* (*Mundinia*) *orientalis* is a newly described human pathogen causing cutaneous leishmaniasis in immunocompetent individuals and disseminated cutaneous leishmaniasis accompanying visceral leishmaniasis in immunocompromised individuals (Jariyapan et al., 2018). To understand the transmission and infection biology of this new pathogen studies on the properties of metacyclic promastigotes are required. For example, analysis of the transcriptome and proteome of *in vitro*-derived metacyclic promastigotes and *in vivo*-derived (from midges and/or sand flies) metacyclic promastigotes to identify genes/proteins involved in the infection process in different vertebrate animals, and adaptations of these to their specific microenvironments. So far, only one study on the cultivation and characterization of *L. orientalis* promastigotes has been performed (Chanmol et al., 2019a), and investigations into metacyclogenesis and suitable culture conditions for mass production of *L. orientalis* metacyclic promastigotes have not been conducted. Therefore, this study aimed to investigate whether by manipulating culture conditions we could develop an *in vitro* system generating *L. orientalis* promastigote cultures with high proportion and numbers of metacyclic promastigotes. Amongst the factors investigated were included: i) the type of culture media, ii) the pH, iii) the percentage of fetal bovine serum (FBS), and iv) the addition of various other media supplements. In addition, purification of *L. orientalis* metacyclic promastigotes from the cultures using peanut lectin agglutination (PNA) was tested to attempt enrichment of metacyclic promastigotes. Metacyclogenesis was functionally assessed by resistance to complement-mediated lysis and *in vitro* infectivity to THP-1 macrophages.

## Materials and methods

### Reagents

Dithiothreitol (DTT; Sigma-Aldrich, St. Louis, MO, USA), Basal Medium Eagle (BME) vitamins (Sigma-Aldrich, St. Louis, MO, USA), and pooled human urine (Innovative Research Inc., Peary Court Novi, MI, USA) were used.

### Parasite strain

*L. orientalis* (MHOM/TH/2014/LSCM4) parasites were originally isolated from a skin sample of an autochthonous cutaneous leishmaniasis patient in Thailand (Jariyapan et al., 2018) and initially cultured in Schneider’s insect medium (SIM) (Sigma-Aldrich, St Louis, MO, USA) supplemented with 20% (v/v) FBS (Life Technologies-Gibco, Grand Island, NY, USA) and 25 μg/ml gentamicin sulfate (Sigma-Aldrich, St Louis, MO, USA). Then, promastigotes were sub-cultured in Medium 199 (Hyclone, Logan, UT, USA), pH 7.0, supplemented with 10% (v/v) FBS and 25 µg/ml gentamicin sulfate (Sigma-Aldrich, St Louis, MO, USA) at 26 °C for two passages. Parasites (~1 × 10^7^ cells/ml) were cryopreserved in 7.5% (v/v) glycerol in this culture medium and stored in liquid nitrogen. Such cryopreserved promastigotes of *L. orientalis* were used to initiate the cultures for this study.

### Human monocytic cell line (THP-1) culture

The human monocytic cell line THP-1 (gift from Dr. Sirida Yangshim, Department of Microbiology, Faculty of Medicine, Chiang Mai University, Thailand) was maintained in RPMI-1640 medium (Hyclone, Logan, UT, USA) supplemented with 10% (v/v) FBS, pH 7.4, at 37°C in a 5% CO_2_ incubator (Chanmol et al., 2019a). The cells were subpassaged every 3 days to prevent cell density from exceeding 1 × 10^6^ cells/ml and to maintain differentiation ability. For macrophage differentiation, phorbol 12-myristate 13-acetate (PMA) was added into the THP-1 cell culture (2.5 × 10^5^ cells/ml) on day 3 at a final concentration of 10 ng/ml (Jain et al., 2012). Then, 200 μl of PMA-treated cells was dispensed to each well of 8-well Lab-Tek culture chamber slides (Nalge Nunc International, NY, USA). The cells were incubated at 37°C in a 5% CO_2_ incubator for 48 h to allow complete differentiation. After the 48 h incubation period, the cells were washed with pre-warmed RPMI-1640 culture medium and then adherent cells were observed before being used for the preparation of promastigotes or infection assays.

#### Preparation of Promastigotes

To prepare consistent and infectious populations of *L. orientalis* promastigotes for experiments parasites were recovered from infected THP-1 macrophages as follows. Cryopreserved promastigotes (~1 × 10^7^ cells/ml) were thawed gently and cultured in M199 medium for two passages. Stationary phase promastigotes were then used to infect THP-1 macrophages in 8-chamber Lab-Tek tissue culture slides (Nalge Nunc International, USA) at a ratio of 10 parasites per 1 macrophage. After 8 h of incubation, non-internalized parasites were removed by washing three times using serum-free RPMI-1640 medium, and infected macrophages were then incubated in RPMI-1640 medium supplemented with 10% (v/v) FBS, pH 7.4, at 37°C in a 5% CO_2_ incubator (Jain et al., 2012). After 72 h of incubation, intracellular amastigotes were isolated from the macrophages by resuspending the host cells in the medium and passing them through a 27-gauge needle for 10 times, then pelleting by centrifugation, resuspending in 5 ml of M199 medium, and incubating at 26°C. After 4 days of incubation, the resulting promastigotes were subpassaged into fresh M199 medium and the promastigote cultures that were subsequently obtained were then used in experiments.

### Optimization of culture media and pH for cultivation of *L. orientalis* metacyclic promastigotes

Three types of culture media were compared for the cultivation of *L. orientalis* metacyclic promastigotes: SIM, M199, and Grace’s insect medium (GIM) (Thermo Fisher Scientific, Loughborough, UK). The culture media were supplemented with 20% (v/v) FBS and 25 μg/ml gentamicin sulfate and pH adjusted to 5.0, 5.5, 6.0, 6.5, or 7.0. All media were filtered using 0.22 μm filter membranes (Merck Millipore Ltd., Ireland) before use. Promastigote cultures were initiated at 1 × 10^6^ cells/ml in 5 ml of medium in 25 cm^2^ culture flasks and incubated at 26°C for a period of 5 days. Parasites were collected daily for quantification of parasite density, percentage of metacyclic promastigotes and percentage of viable parasites. Parasite counts were performed using a haemocytometer (Precicolor HBG, Germany) and density was calculated. To estimate the percentage of metacyclic promastigotes, 5 μL samples of each culture were smeared on a microscope slide. The slide was air-dried, fixed in methanol, and stained with 5% (v/v) of Giemsa’s stain solution. A minimum of 200 parasites were examined randomly at each time point and three morphological criteria were used to identify metacyclic promastigotes: cell body width ≤ 1.5 μm, cell body length < 12.5 μm and flagellar length ≥ two times body length (Chanmol et al., 2019a; Chanmol et al., 2019b). Parasite viability was measured using a Trypan blue dye exclusion test on live parasites. 0.4% (w/v) Trypan blue in saline solution (Gibco 15250061, ThermoFisher Scientific) was mixed 1:1 with a sample of culture, which was then examined by light microscopy. Dead cells stained blue while viable cells showed no staining, remained clear, and were motile. Each culture condition (different medium and pH) was subjected to triplicate runs. The medium and pH conditions found to yield the highest percentage of metacyclic promastigotes were selected for further optimization of metacyclic promastigotes’ cultivation as described below.

### Optimization of additives and concentration of FBS

Cultures were initiated with promastigotes at a density of 1 × 10^6^ cells/ml in 25 cm^2^ culture flasks containing 5 ml of the selected medium supplemented with 10% (v/v) or 20% (v/v) FBS. Various combinations of additives were tested as follows: (1) 100 μM DTT and 1% (v/v) BME (DB); (2) 100 μM DTT and 2% (v/v) pooled human urine (DU); or (3) 100 μM DTT, 1% (v/v) BME and 2% (v/v) pooled human urine (DBU), some of which had proved useful for culture of axenic amastigotes (Chanmol et al., 2019a). Cultures containing only 5 ml of the selected medium and no additives supplemented with 10% (v/v) or 20% (v/v) FBS were used as controls. Parasites from each culture condition were collected at a stationary phase (Day 4) for quantification of parasite density, percentage of metacyclic promastigotes, and percentage of parasite viability as described above, and the number of viable metacyclic promastigotes was calculated. Each culture condition was run in triplicate.

### Lectin-binding agglutination assay

The lectin-binding agglutination method described by Saraiva et al. (2005) with some modifications was used to purify metacyclic promastigotes. Briefly, parasite samples from a test culture condition and a control culture condition (the selected medium supplemented with 10% (v/v) FBS, pH 7.0) were collected at the stationary phase of growth by centrifugation at 2,000*×g*, 20°C for 10 min, washed three times with phosphate-buffered saline (PBS), and resuspended in PBS at 10^8^ cells/mL. These were incubated with 100 μg/mL peanut lectin (Sigma-Aldrich, St Louis, MO, USA) for 30 min at room temperature to purify metacyclic promastigotes. After incubation, cell suspensions were centrifuged at 150*×g* for 5 min and then the non-agglutinated metacyclic promastigotes were collected from the supernatant. Live metacyclic promastigotes were counted immediately using a haematocytometer and the percentage of metacyclic promastigotes was determined.

### Complement-mediated lysis assay

Three populations of promastigotes, exponential phase promastigotes, stationary phase promastigotes, and PNA non-agglutinated promastigotes (purified metacyclic promastigotes), were obtained from the same culture medium, and assays were done at the same time to minimize variability from unwanted sources and provide the most accurate comparison among these populations. The promastigote populations were tested for their resistance to complement-mediated lysis as described by Gamboa et al. (2008) with some modifications. Briefly, promastigotes were incubated at a concentration of 2×10^7^ parasites/ml in 100 μl of PBS (control) or doubling dilutions of human serum (platelet poor human plasma, mycoplasma tested, virus tested, Sigma-Aldrich, St Louis, MO, USA) from 1:1 to 1:128 at 37°C for 30 min. Live parasites were determined using the Trypan blue dye exclusion test. The results were expressed as percentage of viable parasites compared to controls that were exposed to PBS. To compare resistance ability, the concentration of serum causing 50% lysis of promastigotes (EC_50_) was calculated from the dose-response curves of each culture condition and expressed as a ratio.

### *In vitro* Infectivity to THP-1 macrophages

THP-1 macrophages in 8-chamber Lab-Tek tissue culture slides were infected with three promastigote populations grown in the same culture medium, i.e., exponential phase promastigotes, stationary phase promastigotes, and PNA non-agglutinated promastigotes, at a ratio of 10 parasites per 1 macrophage. The parasites were allowed to infect the macrophages for 8 h, and then non-internalized parasites were removed using serum-free RPMI-1640 medium. Cultures of infected macrophages were incubated for 24, 48, or 72 h in RPMI-1640 medium supplemented with 10% (v/v) FBS, pH 7.4, at 37°C in a 5% CO_2_ incubator (Jain et al., 2012). After incubation, the slides were fixed in absolute methanol for 2 min and stained with 5% (v/v) of Giemsa’s stain solution for 30 min. At least 300 macrophages from each time point were counted under a light microscope to determine the percentage of infected macrophages, the average number of amastigotes per macrophage, and infection index (percentage of infected macrophages × average number of amastigotes per macrophage). Experiments for each culture condition were performed in triplicate.

### Statistical analysis

For multiple groups of data, ANOVA followed by Tukey’s test was used and compared between mean ± standard deviation. The data were analyzed for statistical significance using an unpaired Student’s t-test (*P* < 0.05 was considered significant). GraphPad Prism 9.1 software (San Diego, CA, USA) was used to calculate the EC_50_ value corresponding to each sample tested using a sigmoidal dose-response model.

## Results

### Growth and development of *L. orientalis* promastigotes in SIM, M199, and GIM at different pH

**Figure 1:**
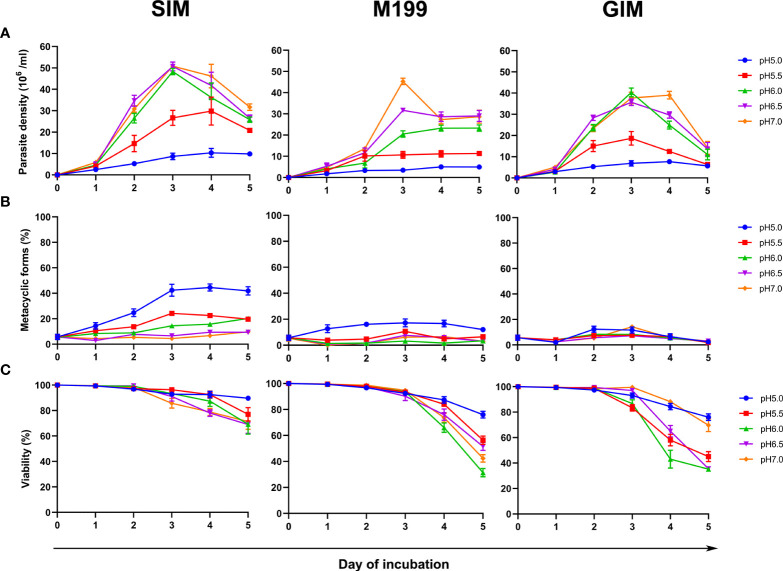
Comparison of density, metacyclogenesis and viability under various culture conditions. Promastigote cultures were initiated at 1 × 10^6^ cells/ml. **(A)** Parasite density, **(B)** Percentage of metacyclic promastigotes, and **(C)** Percentage viability of *L. orientalis* promastigotes cultured in SIM, M199, and GIM at different pH values, 5.0, 5.5, 6.0, 6.5, and 7.0, in each case supplemented with 20% (v/v) FBS. Results are expressed as mean ± standard deviation and are based on three independent replicates.

Three different base media, SIM, M199, and GIM, supplemented with 20% (v/v) FBS were tested for their ability to support the growth of *L. orientalis* metacyclic promastigotes *in vitro* at different pH from 5.0 to 7.0 for a period of 5 days of cultivation. The results revealed that the parasite density, maximum percentage of metacyclic forms, and viability of *L. orientalis* promastigotes cultured in SIM were greater than those cultured in M199 or GIM at every pH tested (**Figure 1**; **Supplementary file 1**). The *L. orientalis* promastigotes cultured in the SIM at pH 6.0-7.0 showed faster growth, reaching approximately 2-3 × 10^7^ cells/ml after day 2, than those cultured at pH 5.5, which reached approximately 2 × 10^7^ cells/ml after day 3, and at pH 5.0, which reached approximately 1 × 10^7^ cells/ml after day 4 (**Figure 1A**; **Supplementary file 1**). However, the highest percentage of metacyclic promastigotes (approximately 44%) was obtained from the SIM culture, pH 5.0, after incubation at 26°C for 4 days with approximately 92% parasite viability (**Figure 1B, C**; **Supplementary file 1**). Therefore, SIM, pH 5.0 was selected for further study.

### Metacyclogenesis of *L. orientalis* in SIM, pH 5.0 supplemented with different additives and concentration of FBS

Subsequently, optimization of combinations of the additives DTT, BME vitamins and human urine, and variation in concentration of FBS added to SIM were examined. Parasite density, percentage of metacyclic promastigotes, and percentage viability of promastigotes in SIM, pH 5.0 with different culture supplements were determined on day 4 of cultivation, as this was at the stationary phase of growth providing the highest percentage of metacyclic promastigotes (**Table 1**). The highest number of the metacyclic promastigotes was obtained from the SIM culture supplemented with DBU and 10% (v/v) FBS (7.17 ± 0.23 × 10^6^ cells/ml), which was statistically significantly different (P < 0.0001) to the SIM culture supplemented with DBU and 20% (v/v) FBS (4.92 ± 0.24 × 10^6^ cells/ml). Regarding purity, the highest % of metacyclic promastigotes was observed in the SIM, DBU, 10% (v/v) FBS culture (63.67 ± 1.53%) (**Table 1**). From these data, we concluded that SIM supplemented with DBU and containing 10% (v/v) FBS was the best of the media tested for stimulation of metacyclogenesis in *L. orientalis*. Upon continuous subpassage in this medium, the percentage of metacyclic promastigotes at the stationary phase declined, to 60% after 3 subpassages and 40% after 6 subpassages. A reduction such as this is invariably seen as a consequence of culture adaption, nevertheless, these densities of metacyclic promastigotes are still quite high and superior to those seen in the other media investigated.

**Table 1:** Parasite density, percentage of metacyclic promastigote, percentage viability, and the number of *L. orientalis* metacyclic promastigotes in SIM, pH 5.0 supplemented with different additives and concentrations of FBS (10% or 20%) on day 4 of cultivation.

Additives in SIM		Density (10^6^ cells/ml)		Metacyclic promastigote (%)		Viability (%)		Number of metacyclic promastigotes (10^6^ cells/ml)	
	% FBS	10	20	10	20	10	20	10	20
Control^a^		10.23 ± 0.35^e^	9.57 ± 0.51	42.67 ± 0.58	47.33 ± 1.53	95.33 ± 2.52	91.67 ± 1.15	4.16 ± 0.15	4.15 ± 0.20
DB^b^		7.23 ± 0.75	9.37 ± 0.32	57.00 ± 4.58	53.67 ± 3.21	93.33 ± 1.15	91.33 ± 1.53	3.86 ± 0.59	4.59 ± 0.37
DU^c^		11.67 ± 0.58	9.57 ± 0.51	42.17 ± 2.47	47.33 ± 2.52	94.67 ± 2.08	90.67 ± 1.53	4.66 ± 0.38	4.11 ± 0.38
DBU^d^		12.47 ± 0.45	10.33 ± 0.58	63.67 ± 1.53	51.67 ± 0.58	90.33 ± 0.58	92.17 ± 1.04	7.17 ± 0.23****^f^	4.92 ± 0.24

a. Control = no other additives except FBS.

b. DB = DTT with BME.

c. DU = DTT with pooled human urine.

d. DBU = DTT with BME and human urine.

e. Mean ± standard deviation based on three independent replicates.

f. P < 0.0001.

### Lectin agglutination assay

**Figure 2:**
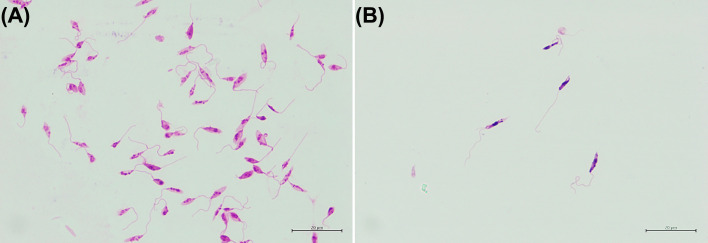
Representative images of **(A)** Stationary phase promastigotes from the SIM, pH 5.0, DBU and 10% (v/v) FBS culture medium and **(B)** PNA non-agglutinated promastigotes.

The lectin peanut agglutinin has previously been shown to selectively agglutinate non-metacyclic promastigote in certain species of *Leishmania* (Saraiva et al., 2005). Suspensions of *L. orientalis* promastigotes cultured in SIM, pH 5.0, DBU and 10% (v/v) FBS and SIM, pH 7.0, and 10% (v/v) FBS were both subjected to lectin agglutination. The initial % of metacyclic promastigotes by morphology in these cultures were 63.67 ± 1.53% (**Table 1**) and 6.67 ± 1.53% (Day 4, **Supplementary file 1**), respectively, but in both cases, peanut lectin agglutination selectively removed non-metacyclic promastigotes. The PNA non-agglutinated population obtained after the lectin agglutination contained purified metacyclic promastigotes (**Figure 2**). The purity of live metacyclic promastigotes by morphology in the resulting PNA non-agglutinated populations were 85.33 ± 2.52% and 81.67 ± 1.53%, respectively. So, although the highest purity (85%) was achieved when starting with a higher initial %, a good purification (82%) could still be achieved from cultures with much lower initial % of metacyclic promastigotes. Thus, PNA selection can be used both as a purification tool and as an assay for metacyclogenesis in *L. orientalis*.

### Complement-mediated lysis

**Figure 3:**
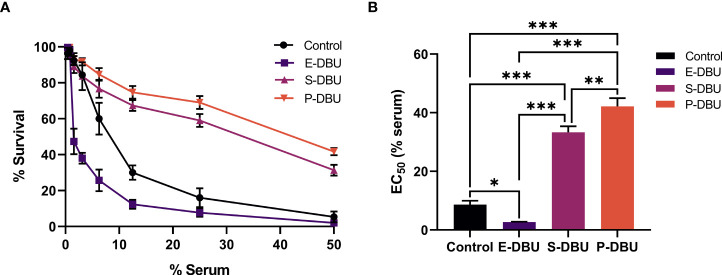
Dose-dependent sensitivity to complement-mediated lysis. **(A)** Percentage survival of exponential phase promastigotes (E-DBU), stationary phase promastigotes (S-DBU), and PNA non-agglutinated promastigotes (P-DBU) cultured in SIM, pH 5.0 with DBU and 10% (v/v) FBS, and stationary phase promastigotes cultured in SIM, pH 7.0 (control) after being exposed to two-fold serially diluted human serum. **(B)** The EC_50_ value of E-DBU, S-DBU, and P-DBU promastigotes cultured in SIM, pH 5.0 with DBU and 10% (v/v) FBS and promastigotes cultured in SIM, pH 7.0 (control). Statistically significant differences between groups are indicated as follows: * *P* < 0.1; ** *P* < 0.01; *** *P* < 0.001.

The populations of promastigotes cultured in SIM, pH 5.0 supplemented with DBU and 10% (v/v) FBS, i.e., exponential phase promastigotes (E-DBU) (day 2), stationary phase promastigotes (S-DBU) (day 4) and PNA non-agglutinated promastigotes (P-DBU) (purified from day 4 S-DBU), and stationary phase promastigotes cultured in SIM, pH 7.0 and 10% (v/v) FBS (control) (day 4) were tested for their ability to resist lysis with normal human serum. Results revealed that in all concentrations of serum tested, the percentage of survival of the P-DBU was the highest and the second highest was of the S-DBU (**Figure 3A**; **Supplementary file 2**). As expected, the percentage of survival of the control (normal stationary phase promastigotes) was higher than that of the E-DBU. The EC_50_ value was calculated from the dose-response curves for each culture medium. The EC_50_ value of the population of the P-DBU was 42.15 ± 2.84 which was statistically higher than that of the S-DBU (33.30 ± 2.07), control (8.66 ± 1.33), and E-DBU (2.72 ± 0.12), respectively (**Figure 3B**; **Supplementary file 3**).

### Infectivity to THP-1 macrophages

**Figure 4:**
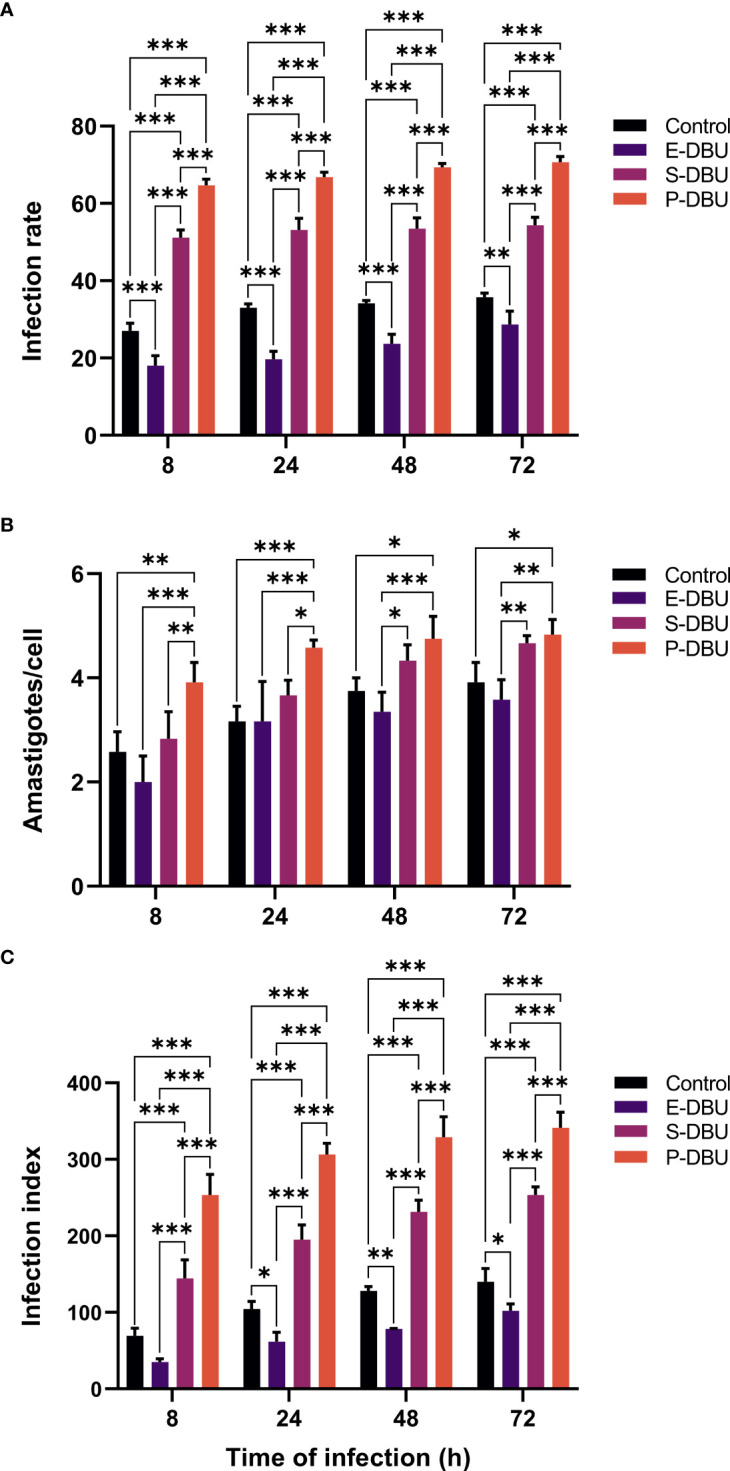
Infection rate, the average number of intracellular amastigotes per cell, and infection index of *L. orientalis* promastigotes for THP-1 macrophages. Exponential phase promastigotes (E-DBU), stationary phase promastigotes (S-DBU), and non-PNA agglutinated promastigotes (P-DBU) cultured in SIM, pH 5.0 with DBU and 10% (v/v) FBS and promastigotes cultured in SIM, pH 7.0 (control) were used to infect THP-1 macrophages. **(A)** Infection rate, **(B)** the average number of intracellular amastigotes per cell, and **(C)** infection index were determined at 8, 24, 48, and 72 h post-infection. Statistically significant differences between groups are indicated as follows: * *P* < 0.1; ** *P* < 0.01; *** *P* < 0.001.

The ability to infect THP-1 macrophages of the E-DBU, S-DBU, and the P-DBU populations cultured in SIM, pH 5.0 supplemented with DBU and 10% (v/v) FBS and stationary phase promastigotes (control) cultured in SIM, pH 7.0 and 10% (v/v) FBS was compared. At all-time points of infection, the infection rate and the infection index of the P-DBU population were significantly higher than those of the S-DBU, the control, and the E-DBU populations, respectively. Also, the infection rate and the infection index of the S-DBU were significantly higher than those of the control, and the E-DBU populations, respectively. At 8 and 24 h of infection, the average number of intracellular amastigotes of the P-DBU population was significantly higher than those of the S-DBU, the control, and the E-DBU populations, respectively, whereas, at 48 and 72 h, it was significantly higher than only those of the control and the E-DBU populations (**Figure 4**; **Supplementary file 4**). The ranges of the infection rates for P-DBU, S-DBU, the control, and E-DBU were approximately 65-71%, 51-54%, 27-36%, and 18-29%, respectively. The ranges of the average number of intracellular amastigotes per macrophage for P-DBU, S-DBU, the control, and E-DBU weres approximately 4-5, 3-5, 2.5-4, and 2-4, respectively. The ranges of the infection index for P-DBU, S-DBU, the control, and E-DBU were approximately 253-341, 145-254, 70-140, and 35-102, respectively (**Supplementary file 4**).

## Discussion

Many types of culture media such as SIM, M199, and GIM have been used for the cultivation of promastigotes of *Leishmania* species with the addition of different supplements depending on specific purposes. Examples of such purposes include initial isolation, diagnosis, mass production, long-term cultivation, vaccine development, generation of axenic amastigotes, and generation of infective parasites (Nayak et al., 2018). For the latter purpose, obtaining high yields of metacyclic promastigotes is a balance between initial stimulation of growth to produce high numbers of promastigotes and stimulation of metacyclogenesis, which is intrinsically growth inhibitory since metacyclic promastigotes are a non-dividing life cycle stage.

In the present study, the use of SIM generated cultures containing approximately 44% of *L. orientalis* metacyclic promastigotes, whereas less than 20% and 10% of metacyclic promastigotes were produced in cultures using M199 and GIM, respectively. The results demonstrated that SIM supported the growth and differentiation to metacyclic stages and viability of *L. orientalis* better than M199 or GIM. These results are consistent with a study in *L. martiniquensis*, another member of the subgenus *L*. (*Mundinia*), in which SIM promoted metacyclogenesis and provided the highest metacyclic ratio (Siripattanapipong et al., 2019). M199 has been used broadly and suggested for mass culture of *Leishmania* parasites (Siripattanapipong et al., 2019). In our study, in M199 cultures the highest parasite density was obtained on day 3, and decreased on days 4 and 5, indicating that M199 supported well the initial growth of *L. orientalis*. For GIM, this medium also supported the growth of *L. orientalis* but not differentiation to metacyclic stages. In a previous study, we have demonstrated that GIM supplemented with 20% (v/v) FBS, 2% (v/v) human urine, 1% (v/v) BME vitamins, and 25 μg/ml gentamicin sulfate, pH 5.5 at 35°C is suitable for cultivation of *L. orientalis* axenic amastigotes (Chanmol et al., 2019a). In this study, differentiation of metacyclic promastigotes cultured in SIM supplemented with DBU and containing 10% (v/v) FBS to axenically cultured amastigotes *in vitro* was also investigated (data not shown). We found that the promastigotes cultured in the medium were transformed into axenic amastigotes when cultured in GIM medium as previously described (Chanmol et al., 2019a).

The real environment that promotes metacyclogenesis of *Leishmania* parasites is in the thoracic midgut (TM) of infected vectors, which are usually sand flies. One important factor appears to be the pH inside the digestive tract of the sand flies. In *Lutzomyia longipalpis*, an important vector of *Leishmania infantum* and a permissive vector of several *Leishmania* species, the pH in the thoracic midgut (TM) of the sand fly is acidic (5.5-6.0), in both unfed flies and during blood digestion, whereas the abdominal midgut (AM) is alkaline during blood digestion (Santos et al., 2008). Our results showed that using SIM, although the lowest parasite density was obtained in SIM at pH 5.0, the highest percentage of metacyclic promastigotes was found indicating that an acidic environment can stimulate metacyclogenesis of *L. orientalis* in culture. These results are in agreement with studies on the stimulation of metacyclogenesis in promastigotes of *L. mexicana*, *L. braziliensis*, *L. donovani*, and *L. major*, where acidification of the medium influenced the development of the parasites towards metacyclic form (Bates and Tetley, 1993; Zakai et al., 1998). Recent evidence has indicated that some or all of the subgenus *Mundinia*, which includes *L. orientalis*, may be transmitted by non-sand fly (midge) vectors (Becvar et al., 2021). If this proves to be true, then it will be interesting to see how the physiology of the midge gut influences metacyclogenesis.

The media supplements DTT, BME vitamins, and human urine stimulated the growth of *L*. *orientalis* and also promoted differentiation to metacyclic promastigotes of *L*. *orientalis*. DTT has been reported that, whilst higher concentrations are harmful, sub-lethal concentrations of hydrogen peroxide can be beneficial in stimulating promastigote to amastigote differentiation in *L. infantum* (Wilson et al., 1994; Khan et al., 2018). Amastigote differentiation is a related but different process to metacyclogenesis, but presumably the beneficial effects of DTT seen here are also mediated by affecting the balance of levels of potentially beneficial and detrimental oxidants.

Vitamins act as co-factors enabling many enzymes to function properly in cells, and in some cases are essential where cells cannot synthesize them, so a vitamin supplement is often required in media. Several reports have shown that 1-5% (v/v) human urine can stimulate the growth of *Leishmania* promastigotes (Howard et al., 1991; Singh et al., 2000; Iqbal et al., 2006; Allahverdiyev et al., 2011). Allahverdiyev et al. (2011) have demonstrated that human urine increased the proliferation and facilitated the transition from G0/G1 to S phases of *Leishmania tropica*, *L. donovani*, and *L. major*, and *L. infantum* parasites more quickly. *Leishmania* are purine auxotrophs, and xanthine has been identified as one component in urine responsible for enhancing the growth of *Leishmania* promastigotes *in vitro* (Warburg et al., 2008). Further, the presence or absence of purines, especially adenosine, influences *Leishmania* metacyclogenesis both *in vitro* and *in vivo* (Serafim et al. (2012). The addition of an adenosine-receptor antagonist to *in vitro* cultures of *Leishmania amazonensis* significantly increased metacyclogenesis, an effect that can be reversed by the presence of specific purine nucleosides or nucleobases. In terms of ingredients in culture media, SIM and GIM do not have purines according to the formulation shown by the manufacturers whereas M199 media according to original formulation contains purines and pyrimidines (Morgan et al., 1950). However, all media were supplemented with FBS and in some cases urine, both of which contain purines. Experiments with defined media will be required to determine the exact substances (or their absence) in SIM that promote metacyclogenesis. Other components in human urine that may play a role in the proliferation and/or differentiation of parasites remain unknown. Howard et al. (1991) reported that urine from each healthy donor enhanced the growth of *L. donovani* and there were slight variations in the growth curves produced by urine samples from different donors. Thus, in the future, it would be interesting to evaluate the stimulation of metacyclogenesis of *L. orientalis* by synthetic human urine (commercially available) using the methods described in this study.

FBS is a complex mixture of various constituents including growth factors, attachment factors, spreading factors, hormones, lipids, sugars, transport proteins, and vitamins, and, although generally used to stimulate growth, contains not only promoters but also inhibitors of growth or differentiation (Gstraunthaler, 2003). It is the most important source of heme, which *Leishmania* cannot synthesize (Chang and Chang, 1985). However, the presence of high amounts of haemoglobin and hemin could inhibit differentiation to metacyclic promastigotes of *Leishmania* parasites and instead stimulate growth of non-metacyclic forms. In that regard, Schlein and Jacobson (1994) have reported that the formation of infective promastigotes of *L. major* is inhibited when haemoglobin or blood is present in its growth medium (Schlein and Jacobson, 1994). Similarly, Charlab et al. (1995) demonstrated that the addition of hemin to the culture medium seems to inhibit the differentiation of *Leishmania amazonensis* metacyclic promastigotes (Charlab et al., 1995). This agrees with our results, where the number of the metacyclic promastigotes of *L. orientalis* found in media containing 10% (v/v) FBS was greater than in media containing 20% (v/v) FBS, a statistically significant difference. An explanation might be that the latter contained an increased amount of haemoglobin, hemin, or other substances that inhibited metacyclogenesis.

The dominant surface glycoconjugate found on the surface of *Leishmania* promastigotes is lipophosphoglycan (LPG), which can bind a variety of lectins (McConville et al., 1992; Soares et al., 2005). Due to developmental changes in LPG structure accompanying differentiation of metacyclic promastigotes, the ability to be agglutinated by certain lectins is lost in metacyclic forms of some species, which allows the isolation of metacyclic promastigotes by negative selection after lectin treatment (da Silva and Sacks, 1987; Almeida et al., 1993; Pinto-da-Silva et al., 2002). Metacyclic promastigotes of *L. major* and *L. donovani* can be purified by negative selection using peanut lectin (McConville et al., 1992; Sacks et al., 1995), whereas, for *Leishmania braziliensis* and *Leishmania guyanensis*, *Bauhinia purpurea* lectin can be used to purify metacyclic promastigotes (Pinto-da-Silva et al., 2002; da Silva et al., 2015; Mendes et al., 2019). In this study, PNA incubation provided an effective way of selectively purifying *L. orientalis* metacyclic promastigotes, even when present at low numbers in cultures, indicating that PNA can be employed as an assay for metacyclogenesis in *L. orientalis*.

Here, two different biological properties that have been associated with metacyclogenesis were examined, namely complement resistance and infection of macrophages. These were used to evaluate and compare metacyclogenesis of *L. orientalis* populations cultured in SIM, pH 5.0, DBU, and 10% (v/v) FBS, the medium that produced the highest yields of metacyclic promastigotes, with stationary phase promastigotes from SIM, pH 7.0 and 10% (v/v) FBS as a control. The results showed that the P-DBU population was more resistant to complement-mediated lysis and infected THP-1 macrophages better than those of the S-DBU, the control, and the E-DBU populations, respectively. These results suggested that the P-DBU population contained the highest amount of metacyclic promastigotes as they were purified from the S-DBU population cultured in the medium and the E-DBU population (day 2) contained less among of metacyclic promastigotes than that of the S-DBU (day 4). It is consistent with the studies in *L. braziliensis* and *L. guyanensis* that total parasites from the log phase (day 2) are less resistant to lysis and *in vitro* macrophage infectivity than those from the stationary phase (day 4 or more) indicating that the log phase populations contain less amount of metacyclic promastigotes that the stationary phase populations (da Silva et al., 2015; Mendes et al., 2019). Moreover, our results indicated that the more metacyclic promastigotes were produced in the pH 5.0 media (in the S-DBU population) than in the pH 7.0 media (in the control population), which was consistent with the results of morphological analysis. Overall, the results show that SIM, pH 5.0 supplemented with DBU, and 10% (v/v) FBS provided a suitable environment supporting the metacyclogenesis of *L. orientalis*.

It is known that the long-term maintenance of *Leishmania* spp. *in vitro* often results in a progressive loss of virulence. For example, in *L. infantum*, a virulence deficit caused by successive *in vitro* passages results from an inadequate capacity to differentiate into amastigote forms (Moreira et al., 2012). Although a loss of virulence through continuous axenic cultivation of *L. orientalis* has not been characterized intensively, in this study, after 6 subpassages the percentage of metacyclic promastigotes was somewhat reduced. Therefore, *L. orientalis* metacyclic promastigotes cultured up to 3 subpassages are recommended for further studies where infectivity is required. Conversely, long term culture may offer a means of attenuation to study genes involved in virulence (Moreira et al., 2012).

As *L. orientalis* is a newly discovered causative agent of human leishmaniasis (Jariyapan et al., 2018), more studies regarding this pathogen are required. Recent studies have demonstrated that experimental infection of guinea pigs with *L. orientalis* produces only temporary erythema and dry lesions at inoculation sites (Becvar et al., 2020) and that metacyclic promastigotes of *L. orientalis* can be transmitted to BALB/c mice by the bites of *Culicoides sonorensis* (Becvar et al., 2021). The true vector(s) of *L. orientalis*, whether they are sand flies and/or midges, are not currently known, but their ability to support metacyclogenesis will be a key determinant in vector incrimination. Also, a suitable animal model of *L. orientalis* to study other aspects of this parasite is not currently available. Experimental infection of other model animals is needed to provide a better understanding of pathogenesis, disease progression, pathology, and drug susceptibility or resistance, and to enable the development of vaccines and drugs for treatment. In addition, studies on the roles of metacyclic promastigotes of *L. orientalis* in infectivity and virulence in different animals and adaptation of the parasite to different microenvironments, analyses of its transcriptome, and proteome as well as gene expression and molecular characterization of the metacyclic promastigotes are required in the future. Thus, this present work would greatly facilitate those aforementioned studies that require the mass production of metacyclic promastigotes from *in vitro* culture.

## Data availability statement

The original contributions presented in the study are included in the article/**Supplementary Material**. Further inquiries can be directed to the corresponding authors.

## Author contributions

Conceptualization, methodology, formal analysis: NJ, WC, KP. Investigation: WC, CM, NJ. Visualization: NJ, KP, CM. Writing - original draft preparation: WC, NJ. Writing-review and editing: NJ, PB, PS. Funding acquisition: NJ, PT, PS. WC and NJ have contributed equally to this work and share the first authorship. All authors contributed to the article and approved the submitted version.

## Funding

This work was supported by Ratchadapiseksompotch Fund, Faculty of Medicine, Chulalongkorn University [grant number RA63/078]; the Program Management Unit for Human Resources & Institutional Development, Research and Innovation - CU [grant number B16F630071]; the TSRI Fund [grant number CU_FRB640001_01_30_1]; and the Faculty of Medicine, Chiang Mai University [grant number 128-2562].

## Acknowledgments

We thank Dr. Sirida Yangshim, Department of Microbiology, Faculty of Medicine, Chiang Mai University, Thailand, for providing THP-1 cells.

## Conflict of interest

The authors declare that the research was conducted in the absence of any commercial or financial relationships that could be construed as a potential conflict of interest.

## Publisher’s note

All claims expressed in this article are solely those of the authors and do not necessarily represent those of their affiliated organizations, or those of the publisher, the editors and the reviewers. Any product that may be evaluated in this article, or claim that may be made by its manufacturer, is not guaranteed or endorsed by the publisher.
