# Supplementary material for: Stimulation of metacyclogenesis in Leishmania (Mundinia) orientalis for mass production of metacyclic promastigotes
Source: Front Cell Infect Microbiol. 2022 Sep 5;12:992741. doi: 10.3389/fcimb.2022.992741 (PMC9483143; doi:10.3389/fcimb.2022.992741)
Supplement: Supplementary file 1 [file Table_1.docx]

**Supplement file 1** Parasite density, percentage of viability, and percentage of metacyclic promastigotes of *L. orientalis* promastigotes cultured in SIM, M199, and GIM at various pH (5.0-7.0) supplemented with 20% FCS, collected from day 1 to 5.

| Day 1 | | | | | | | | | | | | | | | |
| --- | --- | --- | --- | --- | --- | --- | --- | --- | --- | --- | --- | --- | --- | --- | --- |
|  | Density (10^6^ cells/ml) | | | | | Viability (%) | | | | | Metacyclic promastigote (%) | | | | |
| Media | pH | | | | | pH | | | | | pH | | | | |
|  | 5.0 | 5.5 | 6.0 | 6.5 | 7.0 | 5.0 | 5.5 | 6.0 | 6.5 | 7.0 | 5.0 | 5.5 | 6.0 | 6.5 | 7.0 |
| SIM | 2.50±0.50 | 4.17±0.85 | 4.63±0.42 | 4.57±1.52 | 5.87±0.95 | 99.33±0.29 | 99.33±0.29 | 99.33±0.29 | 99.33±0.29 | 99.50±0.00 | 14.33±2.52 | 10.50±0.50 | 8.33±1.26 | 2.83±0.76 | 4.33±0.76 |
| M199 | 1.77±0.25 | 3.27±0.25 | 4.07±0.40 | 5.33±1.50 | 4.73±0.81 | 99.33±0.29 | 99.50±0.00 | 99.33±0.29 | 99.33±0.29 | 99.50±0.00 | 12.67±3.06 | 3.83±0.76 | 1.33±0.29 | 0.83±0.29 | 0.00±0.00 |
| GIM | 2.97±0.55 | 3.17±0.15 | 2.80±0.46 | 4.10±0.60 | 5.07±1.05 | 99.33±0.29 | 99.50±0.00 | 99.33±0.29 | 99.17±0.29 | 99.50±0.00 | 2.00±0.50 | 4.00±1.00 | 4.00±0.50 | 2.33±0.76 | 2.50±0.50 |
| Day 2 | | | | | | | | | | | | | | | |
|  | Density (10^6^ cells/ml) | | | | | Viability (%) | | | | | Metacyclic promastigote (%) | | | | |
| Media | pH | | | | | pH | | | | | pH | | | | |
|  | 5.0 | 5.5 | 6.0 | 6.5 | 7.0 | 5.0 | 5.5 | 6.0 | 6.5 | 7.0 | 5.0 | 5.5 | 6.0 | 6.5 | 7.0 |
| SIM | 5.27±1.17 | 14.67±3.79 | 26.50±2.29 | 34.67±2.57 | 30.83±1.44 | 96.83±1.04 | 97.17±0.29 | 99.00±0.50 | 99.33±0.29 | 98.17±0.62 | 24.67±3.06 | 13.67±1.15 | 8.83±0.58 | 7.67±0.29 | 5.33±0.47 |
| M199 | 3.33±0.58 | 10.17±2.47 | 6.83±2.36 | 11.67±2.25 | 13.67±1.15 | 96.67±1.26 | 97.83±0.76 | 97.83±1.04 | 97.33±0.58 | 98.67±0.47 | 16.10±1.77 | 4.67±0.58 | 1.63±0.15 | 1.63±0.12 | 1.60±0.08 |
| GIM | 5.33±1.15 | 15.00±2.65 | 23.67±1.53 | 28.33±1.26 | 23.33±1.53 | 97.33±0.76 | 98.67±0.58 | 98.83±0.29 | 99.33±0.29 | 98.33±0.47 | 12.33±2.36 | 7.40±0.66 | 8.33±1.15 | 5.67±0.76 | 5.33±0.47 |
| Day 3 | | | | | | | | | | | | | | | |
|  | Density (10^6^ cells/ml) | | | | | Viability (%) | | | | | Metacyclic promastigote (%) | | | | |
| Media | pH | | | | | pH | | | | | pH | | | | |
|  | 5.0 | 5.5 | 6.0 | 6.5 | 7.0 | 5.0 | 5.5 | 6.0 | 6.5 | 7.0 | 5.0 | 5.5 | 6.0 | 6.5 | 7.0 |
| SIM | 8.67±1.53 | 26.67±3.51 | 48.33±1.44 | 50.67±2.08 | 50.83±1.04 | 92.83±1.76 | 96.33±1.53 | 93.33±1.53 | 91.00±3.61 | 85.67±3.79 | 42.33±3.86 | 24.17±0.76 | 14.43±0.51 | 6.50±0.50 | 4.47±0.45 |
| M199 | 3.47±0.65 | 10.67±1.60 | 20.50±1.50 | 31.67±1.15 | 45.33±1.53 | 92.33±1.53 | 93.33±1.53 | 93.67±1.61 | 90.17±3.40 | 94.67±0.58 | 17.17±2.46 | 10.60±0.66 | 3.17±0.38 | 7.60±0.36 | 6.17±0.76 |
| GIM | 7.2±1.28 | 18.67±3.21 | 40.33±2.08 | 35.67±1.53 | 37.67±2.75 | 93.00±2.00 | 83.33±2.52 | 86.67±3.06 | 97.00±1.00 | 99.50±0.00 | 11.67±2.01 | 7.60±0.53 | 8.27±0.25 | 7.23±0.75 | 14.30±1.25 |
| Day 4 | | | | | | | | | | | | | | | |
|  | Density (10^6^ cells/ml) | | | | | Viability (%) | | | | | Metacyclic promastigote (%) | | | | |
| Media | pH | | | | | pH | | | | | pH | | | | |
|  | 5.0 | 5.5 | 6.0 | 6.5 | 7.0 | 5.0 | 5.5 | 6.0 | 6.5 | 7.0 | 5.0 | 5.5 | 6.0 | 6.5 | 7.0 |
| SIM | 10.33±2.08 | 29.33±5.51 | 36.17±6.79 | 41.83±6.17 | 46.17±5.48 | 92.50±2.50 | 92.50±2.78 | 87.33±4.16 | 78.00±2.65 | 78.83±2.75 | 44.50±2.78 | 22.50±0.50 | 15.67±1.15 | 9.33±0.58 | 6.67±0.29 |
| M199 | 5.00±0.82 | 11.17±1.53 | 23.27±1.42 | 28.67±2.25 | 27.33±2.02 | 87.33±2.52 | 84.00±1.73 | 66.00±3.61 | 76.00±4.36 | 73.67±3.51 | 16.67±2.52 | 4.83±0.21 | 1.63±0.15 | 5.90±0.26 | 6.57±0.51 |
| GIM | 7.67±0.95 | 12.47±0.68 | 24.90±1.85 | 29.67±1.53 | 39.00±1.80 | 84.33±2.52 | 58.00±4.58 | 43.00±7.00 | 65.00±4.58 | 88.17±1.76 | 6.50±2.29 | 6.50±0.50 | 5.50±0.50 | 5.00±0.87 | 5.50±0.87 |
| Day 5 | | | | | | | | | | | | | | | |
|  | Density (10^6^ cells/ml) | | | | | Viability (%) | | | | | Metacyclic promastigote (%) | | | | |
| Media | pH | | | | | pH | | | | | pH | | | | |
|  | 5.0 | 5.5 | 6.0 | 6.5 | 7.0 | 5.0 | 5.5 | 6.0 | 6.5 | 7.0 | 5.0 | 5.5 | 6.0 | 6.5 | 7.0 |
| SIM | 9.83±0.76 | 20.83±1.04 | 25.83±1.26 | 26.50±1.50 | 31.67±1.53 | 89.67±1.26 | 77.00±5.29 | 69.17±7.75 | 69.00±6.93 | 71.33±6.11 | 41.83±3.25 | 19.60±0.85 | 20.17±1.03 | 9.33±0.76 | 9.67±0.76 |
| M199 | 94.97±0.85 | 11.33±0.76 | 23.33±1.53 | 29.00±2.65 | 28.67±3.06 | 76.00±2.65 | 56.33±3.21 | 31.33±3.21 | 51.50±3.04 | 42.33±2.89 | 12.00±1.50 | 6.43±0.60 | 3.33±0.31 | 3.03±0.06 | 3.00±0.10 |
| GIM | 5.67±0.76 | 6.33±0.76 | 11.00±2.50 | 13.67±3.06 | 14.17±3.06 | 76.00±2.65 | 45.00±3.97 | 35.17±1.76 | 35.50±1.80 | 69.67±5.03 | 2.33±1.04 | 1.83±0.29 | 2.53±0.37 | 3.00±0.05 | 3.07±0.50 |
