# Supplementary material for: Stimulation of metacyclogenesis in Leishmania (Mundinia) orientalis for mass production of metacyclic promastigotes
Source: Front Cell Infect Microbiol. 2022 Sep 5;12:992741. doi: 10.3389/fcimb.2022.992741 (PMC9483143; doi:10.3389/fcimb.2022.992741)
Supplement: Supplementary file 2 [file Table_2.docx]

**Supplementary file 2** Dose-dependent sensitivity to complement-mediated lysis. Percentage survival of the exponential phase promastigotes (E-DBU), the stationary phase promastigotes (S-DBU), and PNA non-agglutinated promastigotes (P-DBU) cultured in SIM, pH 5.0 with DBU and 10% (v/v) FBS and the stationary phase promastigotes cultured in SIM, pH 7.0 (control) after being exposed to two-fold serially diluted human serum. Results are expressed as mean±standard deviation based on three independent replicates.

| Dilution fold | % Serum | % Survival | | | |
| --- | --- | --- | --- | --- | --- |
|  |  | Control | E-DBU | S-DBU | P-DBU |
| 1:1 | 50.0 | 5.33±3.06 | 2.00±1.00 | 31.33±3.06 | 41.67±2.08 |
| 1:2 | 25.0 | 16.00±5.29 | 7.67±2.31 | 59.00±3.61 | 69.00±3.61 |
| 1:4 | 12.5 | 30.00±4.00 | 12.33±2.52 | 67.33±3.06 | 74.67±3.51 |
| 1:8 | 6.25 | 60.00±8.89 | 25.67±6.03 | 76.67±5.03 | 84.67±3.51 |
| 1:16 | 3.12 | 84.33±1.53 | 38.00±3.00 | 83.67±7.77 | 91.67±2.08 |
| 1:32 | 1.56 | 92.33±2.52 | 47.33±7.09 | 89.00±3.61 | 93.33±3.21 |
| 1:64 | 0.78 | 98.00±1.00 | 98.00±2.00 | 95.33±1.53 | 99.67±0.58 |
| 1:128 | 0.39 | 96.33±3.21 | 99.67±0.58 | 99.00±1.00 | 99.67±0.58 |
