# Supplementary material for: Stimulation of metacyclogenesis in Leishmania (Mundinia) orientalis for mass production of metacyclic promastigotes
Source: Front Cell Infect Microbiol. 2022 Sep 5;12:992741. doi: 10.3389/fcimb.2022.992741 (PMC9483143; doi:10.3389/fcimb.2022.992741)
Supplement: Supplementary file 3 [file Table_3.docx]

**Supplementary file 3** Dose-dependent sensitivity to complement-mediated lysis. EC_50_ value of the exponential phase promastigotes (E-DBU), the stationary phase promastigotes (S-DBU), and PNA non-agglutinated promastigotes (P-DBU) cultured in SIM, pH 5.0 with DBU and 10% (v/v) FBS and the stationary phase promastigotes cultured in SIM, pH 7.0 (control). Results are expressed as mean±standard deviation based on three independent replicates.

| Promastigote population | EC_50_ value |
| --- | --- |
| Control, cultured in SIM, pH 7.0, 10% FBS | 8.66±1.33 |
| E-DBU, cultured in SIM, pH 5.0 supplemented with DBU, 10% FBS | 2.72±0.12 |
| S-DBU, cultured in SIM, pH 5.0 supplemented with DBU, 10% FBS | 33.30±2.07 |
| P-DBU, cultured in SIM, pH 5.0, 10% FBS | 42.15±2.84 |
