# Supplementary material for: Stimulation of metacyclogenesis in Leishmania (Mundinia) orientalis for mass production of metacyclic promastigotes
Source: Front Cell Infect Microbiol. 2022 Sep 5;12:992741. doi: 10.3389/fcimb.2022.992741 (PMC9483143; doi:10.3389/fcimb.2022.992741)
Supplement: Supplementary file 4 [file Table_4.docx]

**Supplement file 4** Infection rate, average number of parasites per macrophage and infection index of the exponential phase promastigotes (E-DBU), the stationary phase promastigotes (S-DBU), and PNA non-agglutinated promastigotes (P-DBU) cultured in SIM, pH 5.0 with DBU and 10% (v/v) FBS and the stationary phase promastigotes cultured in SIM, pH 7.0 (control). Results are expressed as mean±standard deviation based on three independent replicates.

| Promastigote population | Infection rate | | | |
| --- | --- | --- | --- | --- |
|  | Time (h) | | | |
|  | 8 | 24 | 48 | 72 |
| Control, cultured in SIM, pH 7.0, 10% FCS | 27.00±2.00 | 33.00±1.00 | 34.17±0.76 | 35.75±1.42 |
| E-DBU, cultured in SIM, pH 5.0 supplemented with DBU, 10% FBS | 18.00±2.65 | 19.67±2.08 | 23.67±2.52 | 28.67±3.51 |
| S-DBU, cultured in SIM, pH 5.0 supplemented with DBU, 10% FBS | 51.17±2.02 | 53.17±3.01 | 53.50±2.78 | 54.33±2.08 |
| P-DBU, cultured in SIM, pH 5.0, 10% FBS | 64.67±1.61 | 66.83±1.26 | 69.33±1.04 | 70.67±1.53 |
| Promastigote population | Average number of parasites per macrophage | | | |
|  | Time (h) | | | |
|  | 8 | 24 | 48 | 72 |
| Control, cultured in SIM, pH 7.0, 10% FBS | 2.58±0.38 | 3.17±0.29 | 3.75±0.25 | 3.92±0.38 |
| E-DBU, cultured in SIM, pH 5.0 supplemented with DBU, 10% FBS | 2.00±0.50 | 3.17±0.76 | 3.35±0.38 | 3.58±0.38 |
| S-DBU, cultured in SIM, pH 5.0 supplemented with DBU, 10% FBS | 2.83±0.52 | 3.67±0.29 | 4.33±0.31 | 4.67±0.14 |
| P-DBU, cultured in SIM, pH 5.0, 10% FBS | 3.92±0.38 | 4.58±0.14 | 4.75±0.43 | 4.83±0.29 |
| Promastigote population | Infection index | | | |
|  | Time (h) | | | |
|  | 8 | 24 | 48 | 72 |
| Control, cultured in SIM, pH 7.0, 10% FCS | 69.58±9.98 | 104.50±9.99 | 128.00±5.77 | 140.25±17.39 |
| E-DBU, cultured in SIM, pH 5.0 supplemented with DBU, 10% FBS | 35.17±4.48 | 61.67±12.42 | 78.65±0.61 | 102.08±9.06 |
| S-DBU, cultured in SIM, pH 5.0 supplemented with DBU, 10% FBS | 144.54±24.16 | 195.00±19.52 | 231.57±15.21 | 253.50±10.83 |
| P-DBU, cultured in SIM, pH 5.0, 10% FBS | 253.42±27.16 | 306.42±14.75 | 329.13±26.63 | 341.50±20.48 |
